# Temporal Lobe Cavernous Malformation Caused Epileptic Amnesic Episodes and Mild Cognitive Impairment

**DOI:** 10.3389/fneur.2019.00620

**Published:** 2019-06-12

**Authors:** Yusuke Hirokawa, Ayataka Fujimoto, Naoki Ichikawa, Keishiro Sato, Tokutaro Tanaka, Hideo Enoki, Yoshiro Otsuki, Tohru Okanishi

**Affiliations:** ^1^Department of Neurosurgery, Seirei-Hamamatsu General Hospital, Hamamatsu, Japan; ^2^Comprehensive Epilepsy Center, Seirei-Hamamatsu General Hospital, Hamamatsu, Japan; ^3^Department of Pathology, Seirei-Hamamatsu General Hospital, Hamamatsu, Japan

**Keywords:** cavernous malformation, epileptic amnesia, subdural electrode recording, Alzheimer disease-like symptoms, amyloid β, hyperexcitability of the medial temporal lobe

## Abstract

Neuropathological features in Alzheimer's disease (AD) are amyloid β (Aβ) deposits and neurofibrillary changes. AD is characterized by memory impairment and cognitive dysfunction, with some reports associating these impairments with hyperexcitability caused by Aβ in the medial temporal lobe. Epileptic seizures are known to be common in AD. We encountered a 65-year-old patient with cavernous malformation (CM) in the right temporal lobe who exhibited epileptic amnesia (EA) and AD-like symptoms. Scalp electroencephalography (EEG), including long-term video-EEG, showed no interictal discharges, but intraoperative subdural electrode (SE) recording from the right parahippocampal area showed frequent epileptiform discharges. Neuropathologically, senile plaques were found in the surrounding normal cortex of the CM. Postoperatively, the patient has remained free of EA and AD-like symptoms since total removal of the CM. This is the first surgical case report to confirm temporal lobe hyperexcitability associated with EA and AD-like symptoms.

## Background

Alzheimer's disease (AD) is the most frequent neurodegenerative disease causing dementia in elderly people. This pathology is characterized by synapse loss in the cortex, as well as deposition of certain distinctive lesions such as senile plaques and cerebral amyloid angiopathy mainly comprising amyloid β (Aβ) and neurofibrillary tangles composed predominantly of hyperphosphorylated tau (1, 2). Clinically, memory impairment is conspicuous, along with progression of cognitive dysfunctions such as disorientation disorder, aphasia, alexia, or agnosia. On the one hand, incipient protein pathologies such as Aβ in the medial temporal lobe are widely accepted to instigate the loss of episodic memory in AD (3, 4). On the other hand, other investigators have associated these impairments in AD with hyperexcitability of the medial temporal lobe induced by Aβ (5, 6). Epileptic seizures are known to occur in AD (7, 8) and many elderly individuals exhibit temporal lobe epilepsy (9, 10). A link between AD and epilepsy has thus been suggested (11–13).

One report of two cases with AD and amnesia provided a theoretical explanation of amnesic episodes arising from medial temporal lobe epileptic seizures using data from foramen ovale electrodes (12). Here, we encountered a patient with cavernous malformation (CM) in the right temporal lobe who had been diagnosed with AD and who exhibited repetitive and progressive amnesic episodes without epileptiform discharges on scalp electroencephalography (EEG). We hypothesized that the temporal lobe CM had caused the amnesic episodes and AD-like symptoms.

## Case Presentation

**Figure 1:**
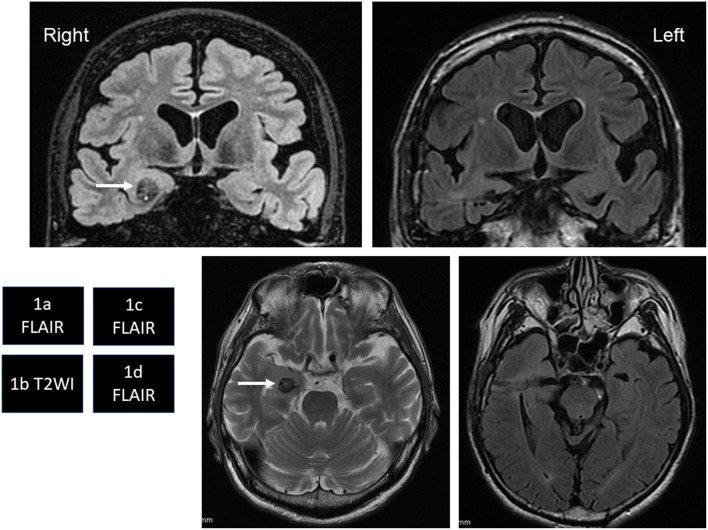
**(a,b)** White arrows in coronal fluid-attenuated inversion recovery (FLAIR) image **(a)** and axial T2-weighted image **(b)** show right temporal cavernous malformation (CM). Cortical atrophy and ventriculomegaly of the brain are also seen. **(c,d)** FLAIR images show total removal of the CM by a transcortical approach.

A 63-year-old, right-handed man exhibited transient episodes of amnesia. He also showed independent, short-duration loss of awareness with oral automatism. He had therefore visited a local hospital, where brain magnetic resonance imaging (MRI) had revealed CM in the right amygdala (Figure 1). As the frequency of loss of awareness and transient amnestic episodes increased and memory disturbances exacerbated over a period of years, he was diagnosed with early-stage AD concomitant with temporal lobe epilepsy at the hospital. He was therefore referred to our epilepsy center at 64 years old.

Seven-day scalp video-EEG performed at our epilepsy center captured no interictal epileptiform discharges, impaired awareness seizures, or oral automatisms. However, based on the clinical history and right amygdala CM, we diagnosed epileptic amnesia (EA) and prescribed levetiracetam. Levetiracetam mildly decreased the frequency of intermittent amnesic episodes, but did not resolve them completely. As we had started speculating that the CM in the right amygdala might have contributed to EA and cognitive deterioration, he underwent neuropsychological examinations: preoperative Mini-Mental State Examination (MMSE) score, 25/30; Hasegawa's dementia scale-revised (HDS-R), 22/30; Weschler memory scale (WMS)-III, verbal memory 79, visual memory 68, total memory 72; trail making test (TMT)-A, 1 min 27 s; TMT-B, 3 min 37 s. We planned minimally invasive intraoperative subdural electrode (SE) recording directly from the parahippocampal area via a small burr hole and small skin incision. We had decided in advance that if epileptiform discharges were obtained from the SE recording, we would proceed to remove the CM. If no epileptiform discharges were obtained, we would just withdraw the SE and close the incision.

**Figure 2:**
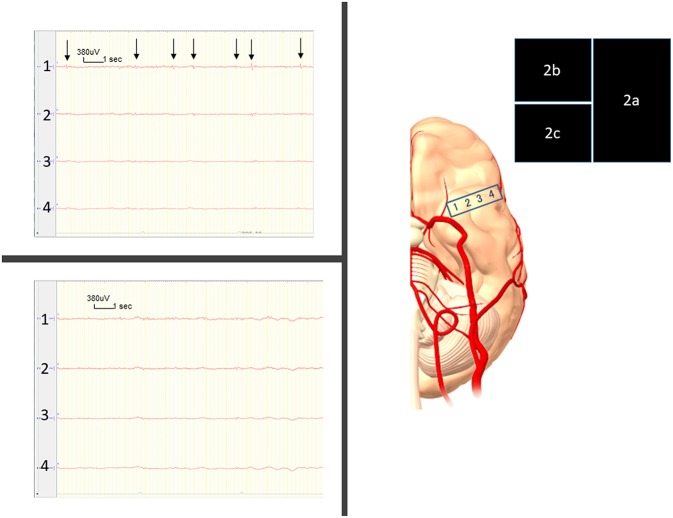
**(a)** Intraoperatively, we place the four-contact subdural strip on the parahippocampal area. **(b)** Frequent, very small-amplitude spikes and spike-waves are seen, mostly from contact 1 (arrows) before removal of the cavernous malformation (CM). **(c)** Right after CM removal, these epileptiform discharges have disappeared.

Intraoperatively, the SE recording from the right parahippocampal area (Figure 2a) showed frequent epileptiform discharges (Figure 2b). We therefore selectively removed the CM under a transcortical approach at 65 years old. After removal of the CM, the SE recording showed no interictal discharges (Figure 2c).

Postoperatively, the patient remained free from transient amnesic episodes and impaired awareness seizures. Four months after the surgery, neuropsychological examinations conducted without changing any medication showed: MMSE, 26/30; HDS-R, 26/30; WMS-R, verbal memory 82, visual memory 68, total memory 74; TMT-A, 42 s; and TMT-B, 2 min 30 s (Table 1). Postoperatively, the patient could walk, talk, and eat faster and became more sociable.

**Table 1:** Differences between pre- and post-CM removal surgery.

	**Transient amnesic episode**	**MMSE**	**HDS-R**	**TMT-A**	**TMT-B**	**WMS-III, visual memory**	**WMS-III, verbal memory**	**WMS-III, total**
Pre CM removal	+	25	22	1 min 27 s	3 min 37 s	68	79	72
Post CM removal	Non	26	26	42 s	2 min 30 s	68	82	74

*CM, cavernous malformation; MMSE, mini mental state examination; HDS-R, Hasegawa's dimentia scale-revised; TMT-A, trail making test-A; TMT-B, trail making test-B; WMS, Weschler memory scale*.

**Figure 3:**
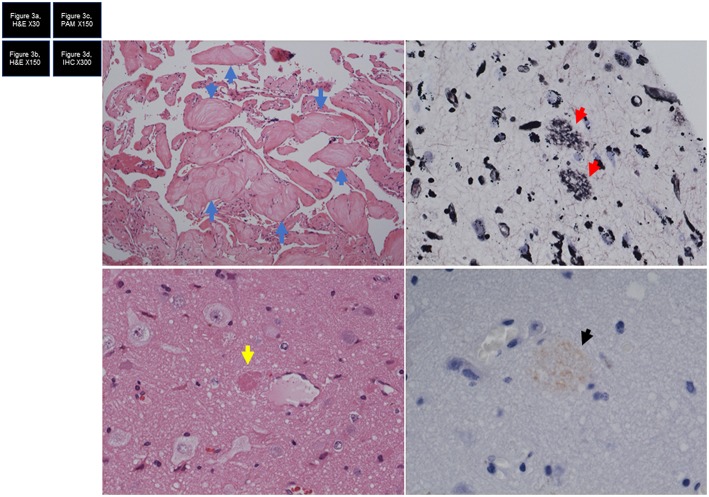
**(a)** Hematoxylin and eosin (HE) staining shows cavernous malformation. Eosinophilic nodules are considered to represent organized thrombi (blue arrow). **(b)** HE staining shows brain tissue with senile plaque (yellow arrow). **(c)** Periodic acid methenamine silver (PAM) staining shows two lesions of filamentous material (red arrows), comprising senile plaque. **(d)** Immunohistochemical staining shows immunoreactive senile plaque (arrow).

The neuropathological examination showed CM, with senile plaques found in the normal cortex surrounding the CM according to periodic acid methenamine silver staining and immunohistochemical staining (Figure 3).

## Discussion

Hyperexcitability of the temporal lobe was directly recorded by the intraoperative SE. Removal of the right amygdala CM completely resolved the transient amnesic episodes and mildly improved patient cognitive function. Therefore, in this case we could say that the CM in the temporal lobe generated medial temporal lobe excitability, as confirmed by the SE recording and that this hyperexcitability produced the EA and AD-like symptoms. Although CM could occur as a *de novo* formation (14, 15), this lesion is generally considered congenital in nature or at least presents as a long-term process (16, 17) and many are found incidentally while the patient is asymptomatic (18–20). Brain hyperexcitability generated by adjacent to CM is widely recognized (21) and this hyperexcitability is associated with epileptogenicity (22, 23). This meant that the patient had been potentially in an epileptic state due to the CM for a long time, but had not exhibited clinical epileptic seizures. We did not regard CM as directly contributing to senile plaque formation, with other causes leading to accumulation of senile plaque in the brain. As senile plaque was found in the patient, the Aβ also added to any underlying hyperexcitability (5, 6, 24) in the potentially epileptic medial temporal lobe with the CM. As his brain MRI showed cortical atrophy and ventriculomegaly, this condition was also attributed to AD-like symptoms.

AD is a complex disorder in which synaptic loss, accumulation of senile plaque, neurofibrillary tangles, and microvascular amyloid is seen in meningocortical regions (2). Overly, this patient had multiple factors causing AD-like symptoms. He had started to develop impaired awareness seizures, EAs, and AD-like symptoms over a period of years. However, removal of the CM reduced the temporal lobe hyperexcitability, and consequently completely eliminated EA and impaired awareness seizures, and mildly improved AD-like symptoms.

We could say that: (1) EA and impaired awareness seizures were generated by temporal lobe hyperexcitability; (2) this temporal lobe hyperexcitability was somehow associated with AD-like symptoms, since the symptoms of EA and impaired awareness seizures completely resolved and symptoms of AD-like symptoms improved mildly after removal of the CM that generated hyperexcitability of the mesial temporal lobe as proven by direct recording.

Memantine works to improve AD as a non-competitive N-methyl-D-aspartate (NMDA) receptor antagonist (25) and its effectiveness against EA has been reported (25, 26). Levetiracetam might improve symptoms of AD (27). A link between epilepsy and dementia with Levy bodies has also been reported (28).

Diagnosing epilepsy in elderly individuals is very difficult, because focal onset impaired awareness seizures are also reportedly short in the elderly (10), while patients with temporal lobe epilepsy may exhibit recent verbal and non-verbal memory dysfunction, cognitive declines (29), and psychiatric symptoms such as fear and hallucinations (30, 31), which are also seen in AD and are hard to distinguish from each other. Moreover, transient EA might not have a phase of impaired awareness seizures (32). The sensitivity and specificity of scalp EEG are low (33, 34). The diagnosis of epilepsy might not be considered until the patient exhibits convulsive seizures.

As epileptic seizures are frequently seen in the earlier stages of AD, which is known as mild cognitive impairment (30), we could say that the patient was in the prodromal state of AD and we were able to cure most of his symptoms. However, we need to follow-up this patient over the long term. Furthermore, accumulation of data from more patients is needed to evaluate whether epilepsy treatment can prevent progression of AD.

As the incidence of epilepsy in elderly patients is increasing dramatically (35), the possibility must be considered that among the many forms of dementia, some underlying conditions associated with epilepsy (36–39) could be curable.

## Data Availability

All datasets generated for this study are included in the manuscript and/or the supplementary files.

## Ethics Statement

Written informed consent for publication of case details was obtained from our patient and his caregiver. This study was approved by the ethics committee at Seirei Hamamatsu General Hospital.

## Author Contributions

YH and AF: acquisition of data. AF, KS, NI, TO, and HE: analysis and interpretation of EEG. AF and NI: surgery. YO: neuropathology. AF, TO, HE, and TT: advice on the paper.

## Contribution to the Field

Cavernous malformation (CM) was found in the right medial temporal lobe (TL) of a 65-year-old man diagnosed with early-onset AD. TL hyperexcitability has been hypothesized as the cause of AD. We therefore thought that the CM might have been the cause of AD symptoms in this patient.

As TL hyperexcitability could not be measured by scalp electroencephalography, we directly measured TL activity near the CM and found epileptic discharges, which meant hyperexcitability in the TL. We therefore removed the CM.

Postoperatively, amnestic episodes disappeared, and AD symptoms were alleviated.

In pathological tissues, CM and senile plaque were observed.

The CM had potential hyperexcitability in the TL over a long period, and Aβ added further hyperexcitability in his old age to the TL, so TL hyperexcitability was created by both CM and Aβ, leading to the clinical symptoms. Among the many forms of dementia, some may involve underlying conditions associated with epilepsy.

### Conflict of Interest Statement

The authors declare that the research was conducted in the absence of any commercial or financial relationships that could be construed as a potential conflict of interest.
